# Tenecteplase in wake-up ischemic stroke trial: Protocol for a randomized-controlled trial

**DOI:** 10.1177/1747493020984073

**Published:** 2021-01-14

**Authors:** Melinda B Roaldsen, Haakon Lindekleiv, Agnethe Eltoft, Mirza Jusufovic, Mary-Helen Søyland, Jesper Petersson, Bent Indredavik, Arnstein Tveiten, Jukka Putaala, Hanne Christensen, Janika Kõrv, Dalius Jatužis, Stefan T Engelter, Gian Marco De Marchis, Tom Wilsgaard, David J Werring, Thompson Robinson, Ellisiv B Mathiesen, Eivind Berge

**Affiliations:** 1 Department of Neurology, University Hospital of North Norway, Tromsø, Norway; 2 Department of Clinical Medicine, UiT The Arctic University of Norway, Tromsø, Norway; 3 Quality and Development Centre, University Hospital of North Norway, Tromsø, Norway; 4 Department of Neurology, Oslo University Hospital, Oslo, Norway; 5 Department of Neurology, Hospital of Southern Norway, Kristiansand, Norway; 6 Department of Neurology, Skåne University Hospital, Malmö, Sweden; 7 Department of Medicine, St. Olavs Hospital, Trondheim University Hospital, Trondheim, Norway; 8 Department of Neurology, Helsinki University Hospital and University of Helsinki, Helsinki, Finland; 9 Department of Neurology, Bispebjerg Hospital and University of Copenhagen, Copenhagen, Denmark; 10 Department of Neurology and Neurosurgery, University of Tartu and Tartu University Hospital, Tartu, Estonia; 11 Department of Neurology and Neurosurgery, Center for Neurology, Vilnius University, Vilnius, Lithuania; 12 Department of Neurology, University Hospital of Basel and University of Basel, Basel, Switzerland; 13 Department of Neurology and Neurorehabilitation, University Department of Geriatric Medicine Felix Platter, University of Basel, Switzerland; 14 Department of Community Medicine, UiT The Arctic University of Norway, Tromsø, Norway; 15 Stroke Research Centre, UCL Queen Square Institute of Neurology, London, UK; 16 Department of Cardiovascular Sciences and NIHR Biomedical Research Centre, University of Leicester, Leicester, UK; 17 Department of Internal Medicine, Oslo University Hospital, Oslo, Norway

**Keywords:** Tenecteplase, wake-up stroke, acute ischemic stroke, intravenous thrombolysis, TWIST

## Abstract

**Background:**

Patients with wake-up ischemic stroke who have evidence of salvageable tissue on advanced imaging can benefit from intravenous thrombolysis. It is not known whether patients who do not fulfil such imaging criteria might benefit from treatment, but studies indicate that treatment based on non-contrast CT criteria may be safe. Tenecteplase has shown promising results in patients with acute ischemic stroke. The aim of the Tenecteplase in Wake-up Ischemic Stroke Trial (TWIST) is to compare the effect of thrombolytic treatment with tenecteplase and standard care versus standard care alone in patients with wake-up ischemic stroke selected by non-contrast CT.

**Methods/design:**

TWIST is an international, investigator-initiated, multi-centre, prospective, randomized-controlled, open-label, blinded end-point trial of tenecteplase (*n* = 300) versus standard care (*n* = 300) in patients who wake up with an acute ischemic stroke and can be treated within 4.5 h upon awakening. Seventy-seven centres in 10 countries (Denmark, Estonia, Finland, Latvia, Lithuania, New Zealand, Norway, Sweden, Switzerland, and the United Kingdom) participate. The primary outcome is the modified Rankin Scale on the ordinal scale (0–6) at three months.

**Discussion:**

TWIST aims to determine the effect and safety of thrombolytic treatment with tenecteplase in patients with wake-up ischemic stroke selected by non-contrast CT.

**Trial registration:**

ClinicalTrials.gov NCT03181360. EudraCT Number 2014-000096-80.

## Introduction and rationale

Thrombolytic treatment with intravenous recombinant tissue plasminogen activator (rt-PA) given within 4.5 h of onset improves clinical outcome after ischemic stroke.^1^ About one in five ischemic strokes occur during sleep,^2^ and these strokes have traditionally been considered ineligible for thrombolytic treatment because the time of onset is unknown. Recent trials have found benefit of intravenous thrombolytic treatment with alteplase in patients with wake-up ischemic stroke (WUS) and mismatch in lesion visibility between diffusion-weighted imaging and fluid attenuation inversion recovery (DWI/FLAIR mismatch) on MRI or signs of penumbra on CT perfusion (CTP).^3,4^ Although thrombolytic treatment has been shown to be effective in patients who fulfil advanced imaging criteria, it is possible that thrombolysis will benefit patients without such radiologic findings as well. Previous studies have shown that DWI/FLAIR mismatch can be absent in as many as 40% of patients with known stroke duration of less than 3 h,^5^ indicating that selection of patients based on advanced imaging criteria could exclude WUS patients who might benefit from thrombolysis. One-third of patients who underwent screening for inclusion in the WAKE-UP trial were excluded because they did not fulfil mismatch criteria.^3^ Previous studies have shown that clinical and radiological findings did not differ between patients with WUS and patients with stroke of known onset within 4.5 h.^6^ The limited availability of emergency MRI and CTP in many hospitals may also prevent patients from receiving treatment. Thrombolytic treatment of WUS selected by non-contrast CT was found to be safe in two prospective, single-armed open-label trials.^7^ A randomized-controlled trial using routinely available brain imaging criteria to select patients for treatment is therefore highly warranted.

Tenecteplase is genetically engineered to have pharmacological advantages over alteplase and has a simpler administration as it is given as a single bolus.^8^ A recent meta-analysis of five randomized controlled trials showed strong evidence of tenecteplase being noninferior to alteplase for acute ischemic stroke.^9^ In one randomized trial, tenecteplase was associated with a higher incidence of reperfusion and improved clinical outcome compared to alteplase.^10^

The aims of TWIST are to answer the following questions:

- Can thrombolytic treatment with tenecteplase given within 4.5 h of waking up with ischemic stroke using non-contrast CT selection criteria improve functional outcome at three months?
- Can findings on non-contrast CT identify patients with wake-up ischemic stroke who benefit from such treatment?

## Methods and design

TWIST is a pragmatic, CT-based prospective, randomized controlled, open-label trial with blinded end-point assessment of intravenous thrombolysis with tenecteplase in patients with acute ischemic stroke upon awakening.

### Research ethics and regulatory approvals

The trial is conducted in accordance with the MRC Guidelines for Good Clinical Practice in Clinical Trials, the Council of Europe’s Convention on Human rights and Biomedicine (CETS No.: 164), the ICH Harmonized Tripartite Guideline for Good Clinical Practice (CPMP/ICH/135/95), and the Declaration of Helsinki (Edinburgh, October 2000). TWIST has received approval from medical research ethical committees and medical agencies in all 10 participating countries. Written, informed consent is obtained from all eligible patients according to approved national regulations.

### Patient population

We aim to include 600 patients (300 in each treatment arm) with WUS who can be treated within 4.5 h after awakening.

### Inclusion and exclusion criteria

Inclusion criteria (simplified)

- Clinical diagnosis of stroke upon awakening (symptoms not present before sleep) with (i) limb weakness and National Institutes of Health Stroke Scale (NIHSS) score ≥3, or (ii) dysphasia.
- Treatment with tenecteplase is possible within 4.5 h of awakening.

Exclusion criteria (simplified)

- Age <18 years.
- NIHSS score >25 or NIHSS consciousness score >2, or seizures.
- Findings on non-contrast CT that indicate the patient is unlikely to benefit from treatment:
   Infarction comprising more than >1/3 of the middle cerebral artery territory.
   Intracranial hemorrhage.
- Active internal bleeding or high risk of bleeding (e.g. major surgery, trauma, gastrointestinal or urinary tract hemorrhage within 21 days, arterial puncture at non-compressible site within 7 days, defect in coagulation, known defect of clotting or platelet function).

The complete list of inclusion and exclusion criteria is shown in Supplemental Material.

### Randomization

Patients are randomized in a 1:1 ratio using a central computer-generated randomization schedule. The schedule employs a minimization algorithm including age, stroke severity (NIHSS), and time since wake-up and is set to balance these characteristics across all centres in all countries.

### Intervention

The intervention group will be given tenecteplase 0.25 mg per kg of body weight (maximum 25 mg), as an intravenous bolus, plus standard care, while the control group will be given standard care without thrombolysis with tenecteplase or any other thrombolytic agent. Both treatment arms will receive best standard care, including intra-arterial interventions for proximal cerebral artery occlusion.

### Clinical and radiological assessments

A timetable of clinical and radiological assessments is shown in Table 1.

**Table 1.** Examinations at baseline and follow-up

	Days							Months
	1					2	7^a^	3
	Time 1^b^	Time 2^c^	30 min	1 h	3 h			
Non-contrast CT	x			x			x	
CT angiography	(x)			x		(x)		
CT perfusion	(x)			(x)				
NIHSS	x					x	x	
BP monitoring	x	x	x	x	x	x		
mRS	x						x	x
Centralized telephone interview								x

Note: Day 1 is the day of entry into the trial.

a Day 7 or day of discharge, whichever occurs first.

b Time 1: at randomization.

c Time 2: at time of intervention/treatment.

BP: blood pressure; NIHSS: National Institutes of Health Stroke Scale; mRS: modified Rankin Scale; (x): optional examination which should not influence the decision to include the patient, unless the results of the examination, according to the judgment of the investigator, show that the patient should or should not receive thrombolytic treatment.

Findings on baseline non-contrast CT that will be assessed are ASPECT Score, presence of early ischemic changes (loss of grey/white matter cortex definition, loss of basal ganglia outline, hypodensity, lesion volume), and hyperdense artery presence and localization.

### Primary efficacy outcome

Functional outcome is defined by the mRS on the ordinal scale (0–6) at three months.

Information on modified Rankin Scale (mRS) at three months is obtained by centralized telephone interview by trained and mRS-certified personnel blinded for allocated treatment.

### Secondary outcomes

Secondary effect outcomes include dichotomized mRS score (0–1 vs. 2–6 and mRS 0–2 vs. 3–6), death from all causes, symptomatic intracranial hemorrhage, any intracranial hemorrhage, major extracranial bleeding, recurrent ischemic stroke, NIHSS and change in NIHSS score from baseline, EuroQol score (EQ-5D-3L), mini-mental status examination score, and health-economic variables at three months, in addition to radiological outcomes at 24 h (see Supplemental Material for complete list).

### Data monitoring body

The Data Monitoring Committee (DMC) is regularly performing unblinded reviews of SAEs in all patients. An independent statistician prepares the data reports. Only the DMC has access to the interim results. If evidence of harm, or evidence of efficacy, the committee will advise the chair of the Steering Committee. The DMC will also be responsible for monitoring the overall conduct of the trial, and may formulate recommendations to improve adherence to protocol, management, procedures, and quality control.

### Sample size estimates

We assume a treatment effect of 10% absolute difference in a binary endpoint setting (mRS 0–1 vs. mRS 2–6) and a distribution between modified Rankin Scale categories similar to that of the WAKE-UP trial^3^ with 42% with favourable outcome in the non-thrombolysed group versus 52% in the thrombolysed group, corresponding to an odds ratio of 1.50. Assuming an effect size specified as an odds ratio of 1.5 from an ordinal logistic regression model and similar distribution of mRS scores in the control group in six levels as in the WAKE-UP trial (categories 5 and 6 merged) of 15%, 27%, 23%, 17%, 13%, and 5%^3^, the estimated sample size of 600 patients yields a power of 80%, with a two-sided significance level of 5%.

### Statistical analyses

We will analyze the data according to the intention-to-treat principle. Functional outcome will be compared between the study groups by means of ordinal logistic regression and adjusted for age, stroke severity (baseline NIHSS), and time since wake-up. In secondary analyses, favourable outcome defined as mRS 0–1 will be compared by means of logistic regression with mRS 2–6, and good outcome defined as mRS 0–2 with mRS 3–6. A separate set of supplementary analyses will be performed stratified by patients who received endovascular treatment and those who did not.

For clinical event outcomes, we will estimate odds ratios and 95% confidence intervals using logistic regression and estimate hazard ratios with corresponding 95% confidence intervals using the Cox proportional hazards model. All analyses will use 5% two-sided level of significance. A detailed statistical analysis plan will be published prior to end of recruitment.

### Study organization and funding

The University Hospital of North Norway is the Sponsor of the trial.

The main source of funding is from the Norwegian Clinical Therapy Research in the Specialist Health Services Research Programme. Additional grants are from the Swiss Heart Foundation, the British Heart Foundation, and the Norwegian National Association for Public Health. The cost of tenecteplase is covered by an unconditional grant from Boehringer Ingelheim Norway KS.

## Discussion

TWIST includes patients with wake-up stroke selected by non-contrast CT and investigates whether these can benefit from intravenous tenecteplase. The effect of thrombolytic treatment in wake-up stroke patients without mismatch criteria on MRI or CTP has not been evaluated in previous randomized controlled clinical trials. Although the rationale for using the specific imaging criteria in the recent clinical trials of reperfusion therapy is well funded theoretically, this cannot be taken as evidence for lack of benefit from treatment in patients without such criteria. DWI/FLAIR mismatch can be absent in 40% of patients with known stroke duration of less than 3 h.^5^ If treatment is offered only to patients fulfilling the imaging criteria of the recent studies, many patients who might benefit from treatment may be excluded. Furthermore, MRI is not available in the emergency setting in many hospitals, and selection based on non-contrast CT may increase access to treatment and reduce delays.

Tenecteplase may potentially improve recanalization compared to alteplase.^11^ The bolus administration and the very rapid onset of action make tenecteplase an attractive option for stroke patients and might possibly reduce the time to recanalization of an occluded cerebral artery compared to alteplase.

We originally based our sample size estimation on the results of a Cochrane systematic review of the effect of rt-PA within 4.5 h of stroke onset,^7^ assessed as a binary endpoint (favourable outcome mRS 0–2 versus mRS 3–6). As the primary endpoint in TWIST is mRS across the full ordinal scale (shift analysis), sample size estimation based on ordinal logistic regression analysis is more appropriate. The revised sample size estimation is based on observations from recent studies on thrombolytic treatment in patients with wake-up stroke.^12,13^ Details are presented in the Supplemental Material. As a result of the revised sample size estimation, the target was increased from 500 to 600 patients. An even larger increase to account for stroke mimics has not been deemed feasible in light of drop in recruitment rate after the onset of the Covid-19 pandemic as well as limited funding.

The TWIST study population is expected to reflect real-life every day clinical practice. If successful, TWIST may substantially increase the proportion of WUS patients eligible for thrombolytic treatment.

## Summary and conclusions

TWIST will show whether patients with wake-up stroke can be treated with tenecteplase within 4.5 h of awakening, and whether non-contrast CT can be used to identify patients who benefit from treatment.

## Supplemental Material

sj-pdf-1-wso-10.1177_1747493020984073 - Supplemental material for Tenecteplase in wake-up ischemic stroke trial: Protocol for a randomized-controlled trial Click here for additional data file. Supplemental material, sj-pdf-1-wso-10.1177_1747493020984073 for Tenecteplase in wake-up ischemic stroke trial: Protocol for a randomized-controlled trial by Melinda B Roaldsen, Haakon Lindekleiv, Agnethe Eltoft, Mirza Jusufovic, Mary-Helen Søyland, Jesper Petersson, Bent Indredavik, Arnstein Tveiten, Jukka Putaala, Hanne Christensen, Janika Kõrv, Dalius Jatužis, Stefan T Engelter, Gian Marco De Marchis, Tom Wilsgaard, David J Werring, Thompson Robinson, Ellisiv B Mathiesen and Eivind Bergenr in International Journal of Stroke
