# Supplementary material for: Tenecteplase in wake-up ischemic stroke trial: Protocol for a randomized-controlled trial
Source: Int J Stroke. 2021 Jan 14;16(8):990–4. doi: 10.1177/1747493020984073 (PMC8554491; doi:10.1177/1747493020984073)
Supplement: sj-pdf-1-wso-10.1177_1747493020984073 - Supplemental material for Tenecteplase in wake-up ischemic stroke trial: Protocol for a randomized-controlled trial [file sj-pdf-1-wso-10.1177_1747493020984073.pdf]

Supplemental Material

Author affiliations:

Melinda B. Roaldsen[1,2], *Haakon Lindekleiv*[3], *Agnethe Eltoft*[1,2], *Mirza Jusufovic*[4], *Mary-Helen Søyland*[5], *Jesper Petersson*[6], *Bent Indredavik*[7], *Arnstein Tveiten*[5], *Jukka Putaala*[8], *Hanne Christensen*[9], *Janika Kõrv*[10], *Dalius Jatužis*[11], *Stefan T Engelter*[12, 13], *Gian Marco De Marchis*[12], *Tom Wilsgaard*[2], *David J Werring*[14], *Thompson Robinson*[15], *Ellisiv B. Mathiesen*[1,2*], *Eivind Berge*[2, 16*†].

1 Department of Neurology, University Hospital of North Norway, Tromsø, Norway

2 Department of Clinical Medicine, UiT The Arctic University of Norway, Tromsø, Norway

3 Quality and Development Centre, University Hospital of North Norway, Tromsø, Norway

4 Department of Neurology, Oslo University Hospital, Oslo, Norway

5 Department of Neurology, Hospital of Southern Norway, Kristiansand, Norway

6 Department of Neurology, Skåne University Hospital, Malmö, Sweden

7 Department of Medicine, St. Olavs Hospital, Trondheim University Hospital, Trondheim, Norway

8 Neurology, Helsinki University Hospital and University of Helsinki, Helsinki, Finland

9 Department of Neurology, Bispebjerg Hospital and University of Copenhagen, Copenhagen, Denmark

10 Department of Neurology and Neurosurgery, University of Tartu and Tartu University Hospital, Tartu, Estonia

11 Department of Neurology and Neurosurgery, Center for Neurology, Vilnius University, Vilnius, Lithuania

12 Department of Neurology, University Hospital of Basel and University of Basel, Basel, Switzerland

13 Neurology and Neurorehabilitation, University Department of Geriatric Medicine Felix Platter, University of Basel, Switzerland

14 Stroke Research Centre, UCL Queen Square Institute of Neurology, London, United Kingdom

15 Department of Cardiovascular Sciences and NIHR Biomedical Research Centre, University of Leicester, Leicester, United Kingdom

16 Department of Internal Medicine, Oslo University Hospital, Oslo, Norway

* shared last authorship

†Deceased Feb 6th 2020

Corresponding author: Ellisiv B. Mathiesen

TWIST Protocol version/date 200917

Trial Sponsor: University Hospital of North Norway, NO-9038 Tromsø, Norway

Telephone number: +47 77626000

Trial Registration

EudraCT Number, 2014-000096-80. ClinicalTrials.gov, NCT03181360.

Registered June 8, 2017.

We report the trial protocol in accordance with the Standard Protocol Items: Recommendations for Interventional Trials (SPIRIT) statement. Please view submitted checklist.

Study organisation

Sponsor

The University Hospital of North Norway, Tromsø, Norway is the Sponsor of the trial.

The Trial Coordinating Centre is based at the University Hospital of North Norway and the Brain and Circulation Research Group at UiT The Arctic University of Norway. The Trial Coordinating Centre consists of the following persons: Trial Coordinating Investigator Ellisiv B. Mathiesen, Trial Manager Melinda B. Roaldsen, Trial officer Agnethe Eltoft, Assistant Trial Manager Mary-Helen Søyland, Trial IT Manager David Perry and Trial Research Nurse Tone Bratteng. Eivind Berge had a central role in the initiation, planning and implementation of the trial and was Trial Co-coordinating Investigator until his death in Feb. 2020.

Trial Statistician: Tom Wilsgaard.

Trial Steering Committee: Bent Indredavik (Chair), Thompson G. Robinson, David Werring, Arnstein Tveiten, Jesper Petersson, Hanne Christensen, Helle Iversen, Jukka Putaala, Janika Kõrv, Dalius Jatuzis, Gian Marco De Marchis, Stefan Engelter, Erik Lundström, Tom Wilsgaard and Ellisiv B. Mathiesen.

Independent Data Monitoring Committee: Terje Pedersen (Chair), Hans Wedel (statistician) and Peter Sandercock. An independent statistician, Ola Løvsletten, produces the unblinded statistical reports for the DMC.

Event Adjudication Committee: Centralized blinded evaluation of all events is performed by Stein-Harald Johnsen (Chair), Michael Mazya and Thomas Christensen.

Patient Advisory Board: Arne Hagen (the Norwegian Association for Stroke Survivors) and Anne Heimdal (LHL Stroke).

Image Analysis Centre: Centralized blinded evaluation of all radiological images is done by Andrew Bivard and Mark Parsons (Melbourne Brain Centre, University of Melbourne, Royal Melbourne Hospital, and the University of New South Wales, Australia).

Supplementary methods

Inclusion and exclusion criteria

Complete list of inclusion criteria

- Stroke symptoms on awakening that were not present before sleep
- Clinical diagnosis of stroke with limb weakness with NIHSS score ≥3, or dysphasia
- Treatment with tenecteplase is possible within 4.5 hours of awakening
- Written consent from the patient, non-written consent from the patient (witnessed by non-participating health care personnel), or written consent from the nearest family member

Complete list of exclusion criteria

- Age <18 years
- NIHSS score >25 or NIHSS consciousness score >2, or seizures during stroke onset
- Findings on plain CT that indicate that the patient is unlikely to benefit from treatment:
    - Infarction comprising more than >1/3 of the middle cerebral artery territory on plain CT or CT perfusion
    - Intracranial haemorrhage, structural brain lesions which can mimic stroke (e.g. cerebral tumour)
- Active internal bleeding or high risk of bleeding, e.g.:
    - Major surgery, trauma or gastrointestinal or urinary tract haemorrhage within the previous 21 days, or arterial puncture at a non-compressible site within the previous 7 days

  - Any known defect in coagulation, e.g., current use of vitamin K antagonist with an INR >1.7 or prothrombin time >15 seconds, or use of direct thrombin inhibitors or direct factor Xa inhibitors during the last 24 hours (unless reversal of effect can be achieved by agents such as idarusizumab) or with elevated sensitive laboratory tests (such as aPTT, INR, platelet count, eucarin clotting time, TT, or appropriate factor Xa activity assays), or heparins during the last 24 hours or with an elevated aPTT greater than the upper limit of normal
  - Known defect of clotting or platelet function or platelet count below 100,000/mm^3 (but patients on antiplatelet agents can be included)
  - Ischaemic stroke or myocardial infarction in previous 3 months, previous intracranial haemorrhage, severe traumatic brain injury or intracranial or intraspinal operation in previous 3 months, or known intracranial neoplasm, arteriovenous malformation or aneurysm
- Contraindications to tenecteplase, e.g., acute bacterial endocarditis or pericarditis; acute pancreatitis; severe hepatic dysfunction, including hepatic failure, cirrhosis, portal hypertension; active hepatitis; systemic cancer with increased bleeding risk; haemostatic defect including secondary to severe hepatic, renal disease; organ biopsy; prolonged cardiopulmonary resuscitation > 2 min (within 2 weeks)
- Persistent blood pressure elevation (systolic ≥185 mmHg or diastolic ≥110 mmHg), despite blood pressure lowering treatment
- Blood glucose <2.7 or >20.0 mmol/L (use of finger-stick measurement devices is acceptable)
- Pregnancy, positive pregnancy test, childbirth during last 10 days, or breastfeeding. In any woman of childbearing potential, a pregnancy test must be performed and the result assessed before trial entry

- Other serious or life-threatening disease before the stroke: severe mental or physical disability (e.g. Mini Mental Status score <20, or mRS score ≥3), or life expectancy less than 12 months
- Patient unavailability for follow-up (e.g. no fixed address)

<u>Standard care</u>

Both the intervention group and the control group should be given best standard care, according to clinical guidelines. This includes intra-arterial interventions for proximal cerebral artery occlusion, when appropriate. If the patient is given tenecteplase, then aspirin or other antiplatelet or anticoagulant drugs shall not be given until 24 hours after termination of infusion and after the control CT brain scan. Patients allocated to control should receive aspirin 300 mg as a loading dose as soon as possible after randomization (unless there are contraindications to aspirin). After first 24 hours the recommended daily dose of aspirin is 75 mg once daily in both the tenecteplase group and the control group. Best standard care during the first week also include treatments to maintain normal homeostasis (temperature, blood glucose, hydration, nutrition), as well as lipid lowering and blood pressure lowering drugs, in accordance with clinical guidelines. Clinical examinations, including additional CT scans should be performed as clinically indicated.

Primary outcome (complete list)

Functional outcome (defined by the mRS) at 3 months

Secondary outcomes (complete list)

Clinical events:

- Favourable functional outcome: mRS 0-1
- Good functional outcome: mRS 0-2
- Death from all cause during follow-up
- Any intracranial haemorrhage during follow-up
- Symptomatic intracranial haemorrhage by SITS-MOST[1] definition
- Symptomatic intracranial haemorrhage by IST-3[2] definition
- Parenchymal haemorrhage type 2[3]
- Stroke progression during follow-up
- Recurrent ischaemic stroke during follow-up
- Major extra cranial bleeding
- NIHSS score at 24 hours and day 7
- Change in NIHSS score from baseline to 24 hours an day 7

Clinical events are defined in the Appendix.

Other clinical outcomes:

NIHSS score, Barthel Index score, EuroQol score, and MMSE scores at 3 months

Radiological outcomes will be defined in a separate imaging protocol.

Health-economic variables:

- Length of hospital stay
- Nursing home care after discharge
- Re-hospitalisations during first 3 months

Protocol amendments

Inclusion and exclusion criteria:

There have been two major amendments: changes to the inclusion and exclusion criteria (Protocol amendment July 4, 2018) and revision of the sample size estimation (Protocol amendment Sept 17, 2020). In the first major amendment, the inclusion criterion was changed from NIHSS score ≥5 to ≥3. The rationale for this is that many patients with wake-up stroke have mild stroke (low NIHSS score) with clinically relevant deficits, and therefore could be included. Further, we allowed inclusion of patients who were to be treated with intra-arterial interventions for proximal cerebral artery occlusion.

Sample size estimation:

We originally based our sample size estimation on the results of a Cochrane systematic review of the effect of rt-PA within 4.5 hours of stroke onset[4], assessed as a binary endpoint (favourable outcome mRS 0-2 versus mRS 3-6). As the primary endpoint in TWIST is mRS across the full ordinal scale (shift analysis), sample size estimation based on ordinal logistic regression analysis is more appropriate. The revised sample size estimation is based on observations from recent studies on thrombolytic treatment in patients with wake-up stroke[5, 6].

In the largest randomized controlled trial on wake-up strokes, WAKE-UP, the difference between thrombolysed and non-thrombolysed patients was 11,5% for a favourable outcome defined as mRS 0-1. A difference of 11,5% was also found in a recent meta-analysis of six observational studies on patients with unknown stroke onset time, where favourable outcome was defines as mRS 0-2. The MRI-based inclusion criteria in WAKE-UP compared to the CT-based inclusion of TWIST could lead to smaller treatment effect in TWIST. We assume a treatment effect of 10% absolute difference in a binary endpoint setting (mRS 0-1 versus mRS 2-6) and a distribution between mRS categories similar to that of the WAKE-UP trial[7] with 42% with favourable outcome in the non-thrombolysed group vs 52% in the thrombolysed

group, which corresponds to an odds ratio of 1.50, and mRS distribution in the control group in six levels (categories 5 and 6 merged) as 15%, 27%, 23%, 17%, 13%, 5%. With a power of 80%, a two-sided significance level of 5%, and an effect size specified as an odds ratio of 1.50 from an ordinal logistic regression model for the ordinal outcome in the control group, the estimated sample size is 600. T, the revised target is to recruit 600 patients, i.e. 300 patients in each arm.

A complete list of amendments is available in the protocol (https://twist.uit.no)

References:

1. Wahlgren N, Ahmed N, Davalos A, Ford GA, Grond M, Hacke W, et al. Thrombolysis with alteplase for acute ischaemic stroke in the safe implementation of thrombolysis in stroke-monitoring study (SITS-MOST): An observational study. *Lancet*. 2007;369:275-282
2. Sandercock P, Wardlaw JM, Lindley RI, Dennis M, Cohen G, Murray G, et al. IST-3. Collaborative group: The benefits and harms of intravenous thrombolysis with recombinant tissue plasminogen activator within 6 h of acute ischaemic stroke (the third international stroke trial (IST-3): A randomised controlled trial. *Lancet*. 2012;379:2352-2363
3. von Kummer R, Broderick JP, Campbell BC, Demchuk A, Goyal M, Hill MD, et al. The Heidelberg bleeding classification: Classification of bleeding events after ischemic stroke and reperfusion therapy. *Stroke*. 2015;46:2981-2986
4. Wardlaw JM, Murray V, Berge E, del Zoppo G, Sandercock P, Lindley RL, et al. Recombinant tissue plasminogen activator for acute ischaemic stroke: An updated systematic review and meta-analysis. *The Lancet*. 2012;379:2364-2372
5. Thomalla G, Simonsen CZ, Boutitie F, Andersen G, Berthezene Y, Cheng B, et al. MRI-Guided Thrombolysis for Stroke with Unknown Time of Onset. *N Engl J Med*. 2018
6. Zhu RL, Xu J, Xie CJ, Hu Y, Wang K. Efficacy and safety of thrombolytic therapy for stroke with unknown time of onset: A meta-analysis of observational studies. *Journal of stroke and cerebrovascular disease*. 2020;29:104742
